# Systematic Analysis of Immune Infiltrates in High-Grade Serous Ovarian Cancer Reveals CD20, FoxP3 and TIA-1 as Positive Prognostic Factors

**DOI:** 10.1371/journal.pone.0006412

**Published:** 2009-07-29

**Authors:** Katy Milne, Martin Köbel, Steven E. Kalloger, Rebecca O. Barnes, Dongxia Gao, C. Blake Gilks, Peter H. Watson, Brad H. Nelson

**Affiliations:** 1 Trev and Joyce Deeley Research Centre, BC Cancer Agency, Victoria, British Columbia, Canada; 2 Department of Anatomical Pathology, Vancouver General Hospital, Vancouver, British Columbia, Canada; 3 Department of Pathology, University of British Columbia, Vancouver, British Columbia, Canada; 4 Department of Biochemistry and Microbiology, University of Victoria, Victoria, British Columbia, Canada; 5 Department of Medical Genetics, University of British Columbia, Vancouver, British Columbia, Canada; New York University School of Medicine, United States of America

## Abstract

**Background:**

Tumor-infiltrating T cells are associated with survival in epithelial ovarian cancer (EOC), but their functional status is poorly understood, especially relative to the different risk categories and histological subtypes of EOC.

**Methodology/Principal Findings:**

Tissue microarrays containing high-grade serous, endometrioid, mucinous and clear cell tumors were analyzed immunohistochemically for the presence of lymphocytes, dendritic cells, neutrophils, macrophages, MHC class I and II, and various markers of activation and inflammation. In high-grade serous tumors from optimally debulked patients, positive associations were seen between intraepithelial cells expressing CD3, CD4, CD8, CD45RO, CD25, TIA-1, Granzyme B, FoxP3, CD20, and CD68, as well as expression of MHC class I and II by tumor cells. Disease-specific survival was positively associated with the markers CD8, CD3, FoxP3, TIA-1, CD20, MHC class I and class II. In other histological subtypes, immune infiltrates were less prevalent, and the only markers associated with survival were MHC class II (positive association in endometrioid cases) and myeloperoxidase (negative association in clear cell cases).

**Conclusions/Significance:**

Host immune responses to EOC vary widely according to histological subtype and the extent of residual disease. TIA-1, FoxP3 and CD20 emerge as new positive prognostic factors in high-grade serous EOC from optimally debulked patients.

## Introduction

Ovarian cancer is the most deadly gynecologic cancer, affecting more than 190,000 women worldwide each year (International Agency for Research on Cancer). Delayed diagnosis and the presence of widely disseminated disease account for the high mortality associated with the disease. Additionally, while a large percentage of patients initially respond well to cytoreductive surgery and standard chemotherapy, the disease usually recurs within 2-5 years as residual tumor cells develop resistance to chemotherapy [1], [2]. Although prognosis is often poor, numerous favorable prognostic indicators have been described, including early stage, low grade and optimal surgical debulking [3], [4].

Several recent studies have analyzed the influence of host immunity on disease prognosis. Tumor-infiltrating CD3+ T cells are strongly associated with favorable prognosis, specifically when CD3+ cells are localized within tumor epithelium [5]-[9]. These findings have been extended to the CD8+ T cell subset in particular [10]-[17], suggesting that cytotoxic T lymphocytes (CTLs) play an important role in the antitumor immune response. Accordingly, other factors associated with CTL responses are also positively associated with survival, including interferon-γ (IFN- γ) [18], [19], the IFN- γ receptor [20], interferon regulatory factor (IRF)-1 [21], IL-18 [22], TNF-α [23], MHC class I [24]-[26], and MHC class I antigen processing machinery [17].

In contrast to CD8+ T cells, several studies have indicated that tumor-infiltrating CD25+FoxP3+ T cells (referred to as regulatory T cells or Tregs) are associated with decreased survival [10], [27]-[29]. Tregs have the ability to suppress proliferation, cytokine production, and cytolytic activity of CD4+ and CD8+ T cells by mechanisms involving cell-to-cell contact and the release of cytokines such as TGF-β [30], [31]. Tregs can also induce an immunosuppressive phenotype in other cell types such as macrophages [32]. Although Tregs have been associated with poor prognosis in many cancers, several exceptions have recently been reported. Leffers et. al. found that FoxP3+ infiltrates in advanced stage EOC were associated with increased survival [14]. Similar findings have been reported in colorectal cancer [33] and lymphoma [34]-[36]. Furthermore, in murine models, FoxP3+ cells can play a positive role in anti-tumor and anti-viral immunity [37], [38]. The precise role of regulatory T cells in cancer outcomes warrants further consideration given that several groups are attempting to enhance tumor immunity by depleting FoxP3+ Tregs from cancer patients [39]-[44], including EOC patients [45].

In addition to Tregs, other cell types reportedly play an immunosuppressive role in EOC. For example, plasmacytoid dendritic cells contribute to immunosuppression by promoting the development or recruitment of interleukin-10-producing CD4+ and CD8+ regulatory T cells [46], [47]. Myeloid dendritic cells (MDCs) impair T cell immunity by expressing B7-H1, a ligand for the inhibitory receptor PD-1 found on T cells [48]. Monocytes and macrophages in the EOC microenvironment can be polarized toward a so-called M2 phenotype, which is typified by the expression of IL-10, TGF-b and scavenger receptors and is thought to promote tumor progression [49], [50], [51]. Under the influence of IL-6 and IL-10, macrophages in EOC can also express B7-H4, which inhibits T cell proliferation [52]. Macrophages also produce CCL22, which promotes Treg recruitment to the tumor environment [32]. Finally, expression of the inflammatory mediator COX-2 in tumor epithelium has been associated with reduced lymphocyte infiltration and poor prognosis in EOC [13], [53].

With the advent of tumor tissue microarray (TMA) technology, a large number of retrospective studies have investigated the relationship between tumor-infiltrating immune cells and prognosis in EOC and other cancers. However, most studies focus on one or a few markers, such that associations between different immunological factors may be missed. Additionally most studies fail to address the different histological subtypes of EOC, which are now recognized to behave as distinct diseases [54]. As a result, there are inconsistencies and unresolved issues in the literature concerning the prognostic significance of different immune cell infiltrates. To address this, we analyzed several large series of EOC tumors, including high-grade serous, endometrioid, clear cell and mucinous subtypes, for the presence of various immune cell infiltrates and inflammatory markers. Our results reveal that high-grade serous tumors have a distinct immunological profile that is strongly associated with patient survival.

## Results

### Intraepithelial T cells and associated functional markers in high-grade serous EOC

We initially investigated the relationship between immune infiltrates and survival in a cohort of 199 high-grade serous EOC patients. We chose to first focus on high-grade serous cases, as the other histological subtypes exhibit distinct biological and clinical properties that are potentially confounding [54], [55]. This initial cohort was restricted to patients who had undergone optimal cytoreduction (i.e., without evidence of macroscopic residual disease). Patient characteristics are shown in Table 1.

**Table 1:** Clinical characteristics of the optimally debulked high-grade serous EOC patient cohort.

Age at surgery (years)	
Mean	61.00
Std dev	11.48
Range	37.59-85.96
Median	60.08
* Overall Survival (years)	
Mean	5.59
Std dev	3.47
Range	0.4-17.4
Median	4.91
Silverberg Grade	
1	0
2	56
3	143
Unknown	0
Stage	
I	49
II	85
III	65
IV	0
Unknown	0
Total number of evaluable patients	199

* There were no deaths due to causes other than ovarian cancer, therefore disease-specific and overall survival were equivalent.

**Figure 1:**
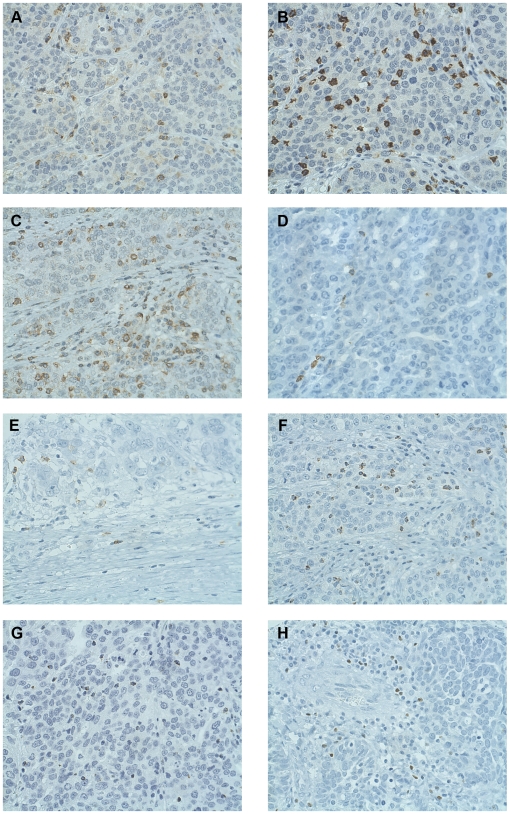
Immunohistochemical analysis of high-grade serous EOC tumors showing infiltrates expressing markers of T cell differentiation and activation. (A) CD4, (B) CD8, (C) CD45RO, (D) OX40, (E) CD25, (F) TIA-1, (G) Granzyme B, and (H) FoxP3. 40X objective.

The tumors in this initial cohort had been previously assessed by immunohistochemistry (IHC) for a variety of lymphocyte markers, including CD3, CD4, CD8, CD20 and Granzyme B [12]. Intraepithelial lymphocytes (i.e., lymphocytes within the epithelial component of the tumor) were scored as either present (i.e. one or more intraepithelial lymphocytes present in at least one of two 0.6 mm cores) or absent. We re-analyzed this data focusing exclusively on high-grade serous cases. We found that 83.2% (163/196) of evaluable high-grade serous tumors were positive for intraepithelial CD3+ T cells, whereas CD4+ and CD8+ intraepithelial cells were found in 53.4% (103/193) and 84.0% (163/194) of evaluable tumors, respectively (Fig. 1A&B and data not shown). CD4+ and CD8+ cellular infiltrates showed a strong positive association (p<0.0001). Table 2 shows statistical associations for these and all other markers studied in this initial cohort.

**Table 2:** *p*-values for Chi-square tests of associations between immunohistochemical markers in the optimally debulked high-grade serous EOC cohort.

	CD3	CD8	CD4	CD45R0	CD25	OX40	TIA-1	GrB^1^
**CD3**		<0.0001	<0.0001	<0.0001	<0.0001	0.16	<0.0001	<0.0001
**CD8**	<0.0001		<0.0001	<0.0001	<0.0001	0.15	<0.0001	<0.0001
**CD4**	<0.0001	<0.0001		0.0021	<0.0001	0.049	0.0013	0.0001
**CD45RO**	<0.0001	<0.0001	0.0021		<0.0001	0.031	<0.0001	<0.0001
**CD25**	<0.0001	<0.0001	<0.0001	<0.0001		0.0040	<0.0001	<0.0001
**OX40**	0.16	0.15	0.049	0.031	0.0040		0.012	0.044
**TIA-1**	<0.0001	<0.0001	0.0013	<0.0001	<0.0001	0.012		<0.0001
**GrB** 1	<0.0001	<0.0001	0.0001	<0.0001	<0.0001	0.044	<0.0001	
**FoxP3**	<0.0001	<0.0001	<0.0001	<0.0001	<0.0001	0.47	<0.0001	<0.0001
**MHC I** 2	<0.0001	<0.0001	<0.0001	<0.0001	<0.0001	0.18	<0.0001	0.0001
**MHC II** 3	0.0078	0.0009	0.013	0.0004	0.0001	0.38	0.0006	0.016
**CD20**	<0.0001	<0.0001	0.0006	0.0036	0.0006	0.13	<0.0001	<0.0001
**CD1a**	0.11	0.096	0.046	0.21	0.27	0.030	0.31	0.48
**CD68**	<0.0001	0.0001	0.0028	0.0019	0.0006	0.23	0.0005	0.0007
**MPO** 4	0.89	0.53	0.034	0.90	0.78	0.33	0.67	0.49
**COX-2** 5	0.75	0.64	0.55	0.64	0.74	0.76	0.42	0.70
	**FoxP3**	**MHC I** 2	**MHC II** 3	**CD20**	**CD1a**	**CD68**	**MPO** 4	**COX2** 5
**CD3**	<0.0001	<0.0001	0.0078	<0.0001	0.11	<0.0001	0.89	0.75
**CD8**	<0.0001	<0.0001	0.0009	<0.0001	0.096	0.0001	0.53	0.64
**CD4**	<0.0001	<0.0001	0.013	0.0006	0.046	0.0028	0.034	0.55
**CD45RO**	<0.0001	<0.0001	0.0004	0.0036	0.21	0.0019	0.90	0.64
**CD25**	<0.0001	<0.0001	0.0001	0.0006	0.27	0.0006	0.78	0.74
**OX40**	0.47	0.18	0.38	0.13	0.030	0.23	0.33	0.76
**TIA-1**	<0.0001	<0.0001	0.0006	<0.0001	0.31	0.0005	0.67	0.42
**GrB** 1	<0.0001	0.0001	0.016	<0.0001	0.48	0.0007	0.49	0.70
**FoxP3**		<0.0001	<0.0001	0.0009	0.35	0.0001	0.42	0.82
**MHC I** 2	<0.0001		<0.0001	0.0015	0.13	0.035	0.61	0.17
**MHC II** 3	<0.0001	<0.0001		0.023	0.11	0.22	0.39	0.30
**CD20**	0.0009	0.0015	0.023		0.87	0.029	0.60	0.13
**CD1a**	0.35	0.13	0.11	0.87		0.52	0.046	0.84
**CD68**	0.0001	0.035	0.22	0.029	0.52		0.43	0.30
**MPO** 4	0.42	0.61	0.39	0.60	0.046	0.43		0.51
**COX-2** 5	0.82	0.17	0.30	0.13	0.84	0.30	0.51	

1 GrB  =  Granzyme B.

2 MHC I  =  MHC class I.

3 MHC II  =  MHC class II.

4 MPO  =  myeloperoxidase.

5 COX-2  =  Cyclooxygenase-2.

While the above markers indicate which lymphocyte subsets are present in tumors, they do not reveal their activation state. To address this issue, we analyzed tumors for expression of CD45RO, OX40 and CD25, which are expressed by activated T cells [56], [57]. Using the same scoring criteria as above, 70.6% (132/187) of tumors were positive for intraepithelial CD45RO+ cells, and 49.7% (96/193) were positive for intraepithelial CD25+ cells (Fig. 1C&E). By contrast, only 7.0% (11/158) of tumors were positive for intraepithelial OX40+ cells (Fig. 1D). In pair-wise comparisons, CD45RO, CD25 and OX40 were all positively associated (Table 2). Moreover, CD45RO and CD25 were both associated with the presence of CD3+, CD4+ and CD8+ cells. OX40 showed a similar trend, but this did not reach statistical significance, likely due to the low number of positive cases.

To investigate the differentiation state of tumor-infiltrating T cells, tissues were analyzed for intraepithelial cells expressing TIA-1 and Granzyme B, which are markers of CD8+ cytotoxic T cells and NK cells [58]-[60]. A majority of tumors (66.5%, 127/191) were positive for intraepithelial TIA-1+ cells (Fig. 1F), and about half of tumors (45.6%, 88/193) were positive for intraepithelial Granzyme B+ cells (Fig. 1G). There was a highly significant association between TIA-1+ and Granzyme B+ cells (p<0.0001). Moreover, in pair-wise comparisons, TIA-1+ and Granzyme B+ cells were each associated with the activation markers CD45RO, CD25 and OX40. Finally, TIA-1+ and Granzyme B+ cells were each associated with the presence of CD3+, CD4+ and CD8+ cells (Table 2). To examine whether TIA-1 and Granzyme B expression could be due to the presence of NK or NKT cells, we examined tumors for the NK cell markers CD56 and CD57. For both markers, there were either few or no infiltrates at all within the tumor epithelium (data not shown), indicating that the TIA-1+ and Granzyme B+ infiltrates were most likely T cells.

Finally, tumors were analyzed for the presence of intraepithelial cells expressing FoxP3, which in humans is a marker of regulatory T cells and activated T cells [61], [62]. About half of tumors (52.9%, 100/189) were positive for intraepithelial FoxP3+ cells (Fig. 1H). There was a strong association between FoxP3+ and CD25+ cells (p<0.0001), and FoxP3+ and CD25+ cells were each strongly associated with CD4+ cells (p<0.0001 for both markers). Thus, consistent with previous reports [10], [14], [27], [28], a significant proportion of tumors contained intraepithelial infiltrates with markers characteristic of Tregs (CD4+,CD25+, and FoxP3+).

### MHC class I and II in high-grade serous EOC

**Figure 2:**
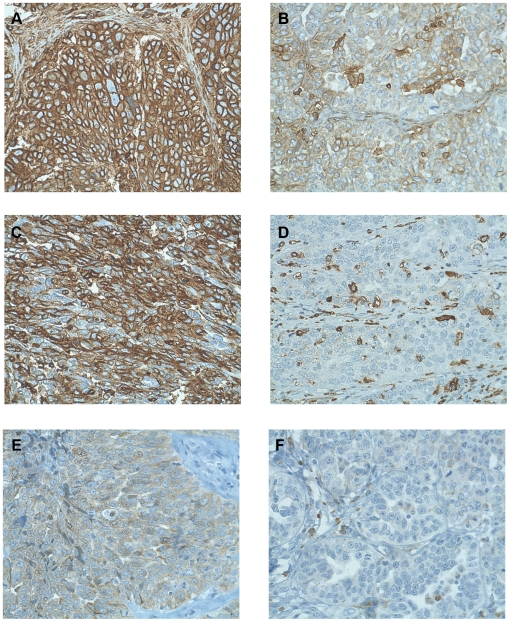
Immunohistochemical analysis of high-grade serous EOC tumors showing (A,B) high and low expression of MHC class I, (C,D) high and low expression of MHC class II, and (E,F) high and low expression of COX-2. 40X objective.

We analyzed tumor cells for expression of MHC class I and II using a four-point scale (negative, focal [<10%], patchy [10-50%] or diffuse [>50%]). All evaluable tumors (185/185) expressed MHC class I to some degree (i.e., focal, patchy or diffuse), indicating they could theoretically present antigen to CD8+ T cells. For statistical analyses, only the highest category (diffuse, >50%) was considered positive (Fig. 2A&B). Using this threshold, 85.4% (158/185) of tumors were positive for MHC class I. MHC class I was positively associated with all three T cell subsets (CD3, CD4, and CD8), the activation markers CD45RO and CD25, and the differentiation markers TIA-1, Granzyme B and FoxP3 (Table 2).

A large majority of tumors (86.5%, 166/192) expressed MHC class II to some degree (i.e., focal, patchy or diffuse), indicating they could theoretically present antigen to CD4+ T cells. As with MHC class I, only the highest category (diffuse, >50%) was considered positive for statistical analyses (Fig. 2 C&D). Using this threshold, 41.1% (79/192) of tumors were positive for MHC class II. MHC class II was strongly associated with MHC class I (p<0.0001). Accordingly, MHC class II was positively associated with all three T cell subsets (CD3, CD4, and CD8), the activation markers CD45RO and CD25, and the differentiation markers TIA-1, Granzyme B and FoxP3 (Table 2). Similar to the results for MHC class I, the expression of MHC class II in tumor epithelium was positively associated with various T cell markers, including CD3, CD4, CD8, CD45RO, TIA-1, Granzyme B, CD25 and FoxP3 (Table 2).

### Intraepithelial B cells in high-grade serous EOC

**Figure 3:**
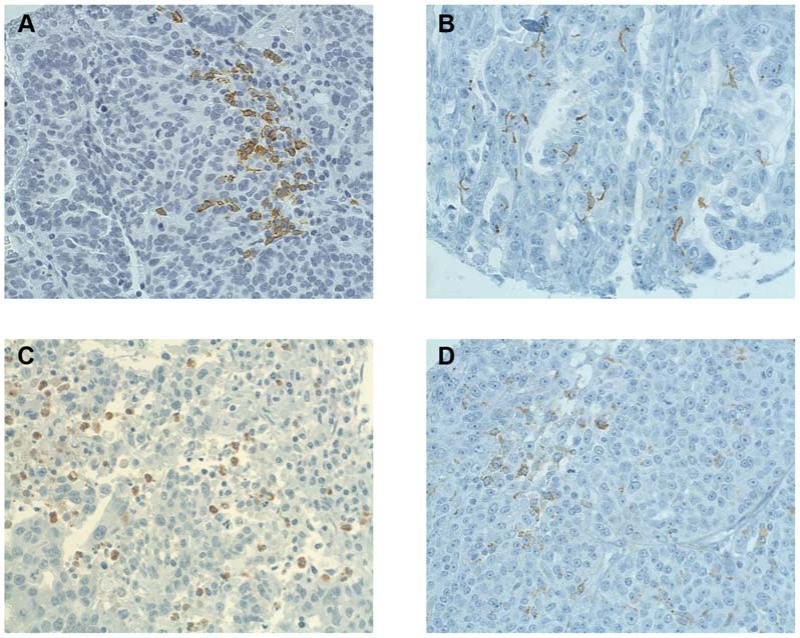
Immunohistochemical analysis of high-grade serous EOC tumors showing infiltrates expressing (A) CD20 (B cells), (B) CD1a (immature DCs), (C) Myeloperoxidase (granulocytes), and (D) CD68 (macrophages). 40X objective.

Tissues were stained with an antibody to CD20, which is expressed by B cells from the naïve to memory stages of differentiation [63]. Intraepithelial CD20+ cells were found in 41.9% (83/198) of evaluable tumors (Fig. 3A). CD20+ infiltrates were strongly associated with all three T cell subsets (CD3, CD4, and CD8); the activation markers CD45RO and CD25; the differentiation markers TIA-1, Granzyme B and FoxP3; and both MHC class I and II (Table 2).

### Intraepithelial dendritic cells, granulocytes and macrophages in high-grade serous EOC

Tumors were analyzed for the presence of immature and mature dendritic cells by staining for CD1a and CD208, respectively. A minority of tumors (13.4%, 23/172) contained intraepithelial CD1a+ cells (Fig. 3B). No significant association with any of the intraepithelial lymphocyte markers (CD3, CD8 or CD20), activation markers (CD45RO or CD25), differentiation markers (TIA-1, Granzyme B or FoxP3) or MHC class I or II (Table 2) was seen, potentially due to the low number of CD1a+ cells. In contrast to CD1a, none of the tumors scored positive for intraepithelial CD208+ cells. Parallel analysis of tonsil tissue revealed the presence of many CD208+ cells, thereby validating the IHC procedure.

About half of tumors (54.7%, 87/159) contained intraepithelial cells expressing the macrophage marker CD68 (Fig. 3D). CD68 was positively associated with several lymphocyte markers (CD3, CD8 and CD20), activation markers (CD45RO and CD25), differentiation markers (TIA-1, Granzyme B and FoxP3) and MHC class I (Table 2). To assess the presence of granulocytes, the TMA was stained for myeloperoxidase. Twenty four percent (37/154) of tumors contained myeloperoxidase-expressing cells (Fig. 3C), however these showed no significant associations with other markers, with the exception of CD4 (p = 0.034).

The COX-2 enzyme has been associated with inferior survival in EOC when expressed in the epithelial component of the tumor [53]. Therefore, tumors were scored for expression of COX-2 in the epithelial component using a four-point scale (negative, equivocal [0-1%], patchy [1-50%] or diffuse [>50%]) (Fig. 2E&F). Two-thirds of tumors (66.5%, 111/167) were positive for COX-2 (i.e., patchy or diffuse staining). In contrast to reports in ovarian, cervical, and other cancers, [13], [64], [65], the expression of COX-2 was not significantly associated with any of the immune infiltrates studied (Table 2).

### Associations between immune infiltrates and patient survival in high-grade serous EOC

**Figure 4:**
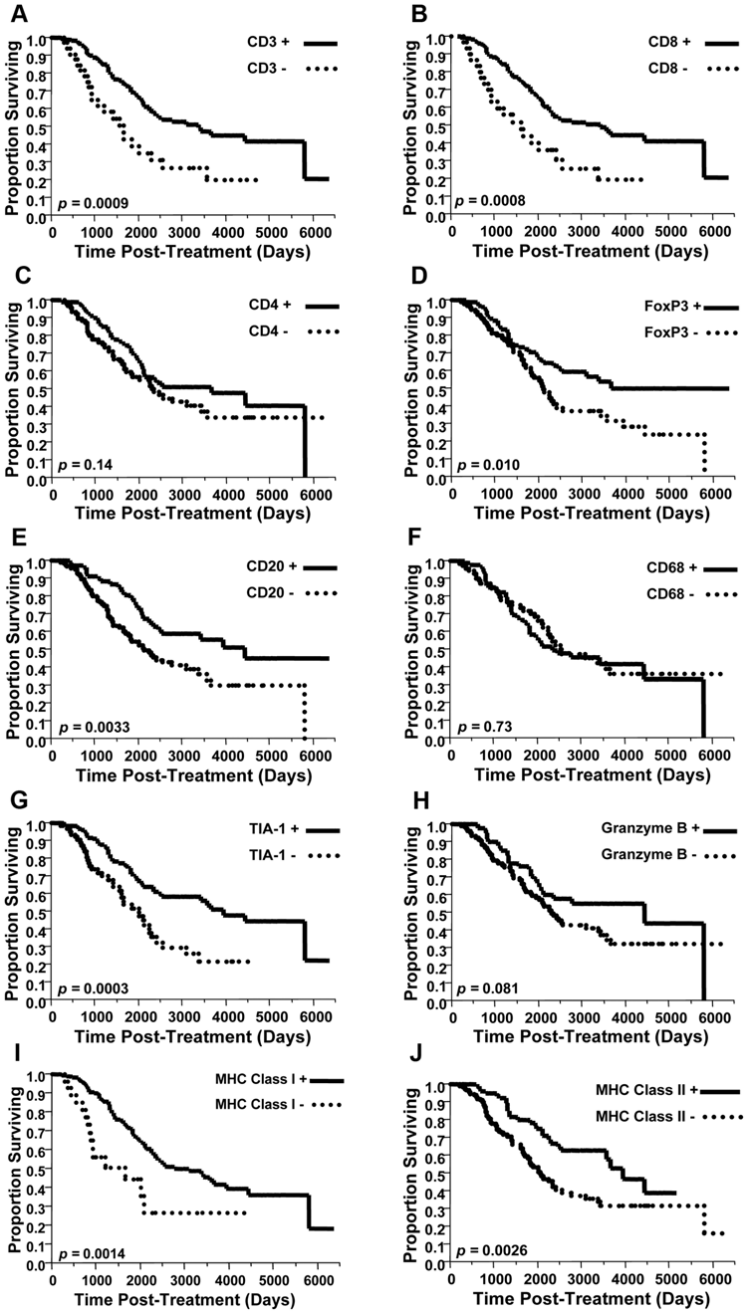
Immune infiltrates and survival in ovarian cancer. Kaplan-Meier curves showing disease-specific survival for patients scored as positive or negative for (A) CD3, (B) CD8, (C) CD4, (D) FoxP3, (E) CD20, (F) CD68, (G) TIA-1, (H) Granzyme B, (I) MHC Class I and (J) MHC Class II. Data were derived from optimally debulked patients with high-grade serous EOC.

Kaplan-Meier analysis was performed to assess the association between various immune infiltrates and disease-specific survival (DSS). Consistent with prior reports [5]-[7], [10]-[17], intraepithelial CD3+ and CD8+ cells were associated with increased DSS (p = 0.0009 and 0.0008 respectively) (Fig. 4A&B). Intraepithelial CD4+ cells showed a trend towards increased DSS, but this was not statistically significant (Fig. 4C). The NK cell markers CD56 and CD57 showed no association with DSS (data not shown).

Intriguingly, intraepithelial CD20+ cells were associated with increased DSS (p = 0.0033) (Fig. 4E). Furthermore, the combination of CD8+ and CD20+ infiltrates was associated with significantly increased DSS over tumors that contained CD8+ infiltrates but not CD20+ infiltrates (median 4432 days vs. 2279 days, p = 0.0115) (data not shown).

In contrast to lymphocyte markers, the dendritic cell marker CD1a showed no association with DSS, possibly due to the low number of tumors containing CD1a+ cells. Likewise, the markers CD68, COX-2 and myeloperoxidase showed no association with DSS (Fig. 4F and data not shown).

Given the association between CD8+ T cells and DSS, we evaluated other canonical features of active CTL responses. DSS was positively associated with intraepithelial TIA-1+ cells (p = 0.0003), as well as expression of MHC class I and II by tumor cells (p = 0.0014 and 0.0026 respectively) (Fig. 4G,I,&J). Tumors that contained both CD8+ and TIA-1+ infiltrates were associated with increased DSS compared to CD8+ TIA-1-negative tumors (p = 0.0025). Several other T cell markers, including Granzyme B, CD45RO and CD25, showed trends toward increased DSS but did not reach statistical significance (Fig. 4H and data not shown). OX-40 showed no apparent trend or association with DSS, possibly due to low numbers of positive cases (data not shown).

In apparent contrast to reports that regulatory T cells are associated with poor prognosis, the presence of intraepithelial FoxP3+ cells was associated with increased DSS (p = 0.010) (Fig. 4D). Moreover, tumors that were triply positive for intraepithelial CD4+, FoxP3+ and CD25+ cells showed a trend towards increased survival, although this fell short of statistical significance (p = 0.059). Likewise, tumors positive for both intraepithelial CD8+ and FoxP3+ cells showed a trend toward increased DSS compared to tumors that were positive for CD8+ cells but negative for FoxP3+ cells; however, this trend did not reach statistical significance (p = 0.052). Thus, by multiple analyses, tumor-infiltrating FoxP3+ cells showed a trend or statistically significant association with increased DSS.

### The association between immune infiltrates and survival is dependent on the extent of residual disease

T cell infiltrates are reportedly more prevalent in patients with optimal versus suboptimal cytoreduction [5], [66]. To investigate whether this was true for other lymphocyte markers, we analyzed an additional cohort of 220 high-grade serous cases from patients known to have macroscopic residual disease following primary cytoreductive surgery. We focused on CD8+ infiltrates, as well as the three novel prognostic markers from the preceding analysis (i.e., FoxP3, TIA-1 and CD20). Compared to the optimally debulked patient cohort, patients with macroscopic residual disease had a significantly lower prevalence of CD8+ (58.5%), FoxP3+ (20.2%), TIA-1+ (39.5%) and CD20+ (16.3%) infiltrates (p<0.0001 for all markers). In Kaplan-Meier analysis of these four markers, only CD8+ infiltrates had a significant association with survival (p = 0.0044) in patients with macroscopic residual disease (data not shown).

### The association between immune infiltrates and survival is dependent on histological subtype

The preceding results were based exclusively on high-grade serous EOC cases. To assess the association between immune infiltrates and DSS in other histological subtypes of EOC, we performed the same analyses using an additional 288 EOC tumors of the following histological subtypes: mucinous (n = 31), endometrioid (n = 125) and clear cell (n = 132). These additional tumor specimens were from a previously described cohort of optimally debulked patients [12].

**Figure 5:**
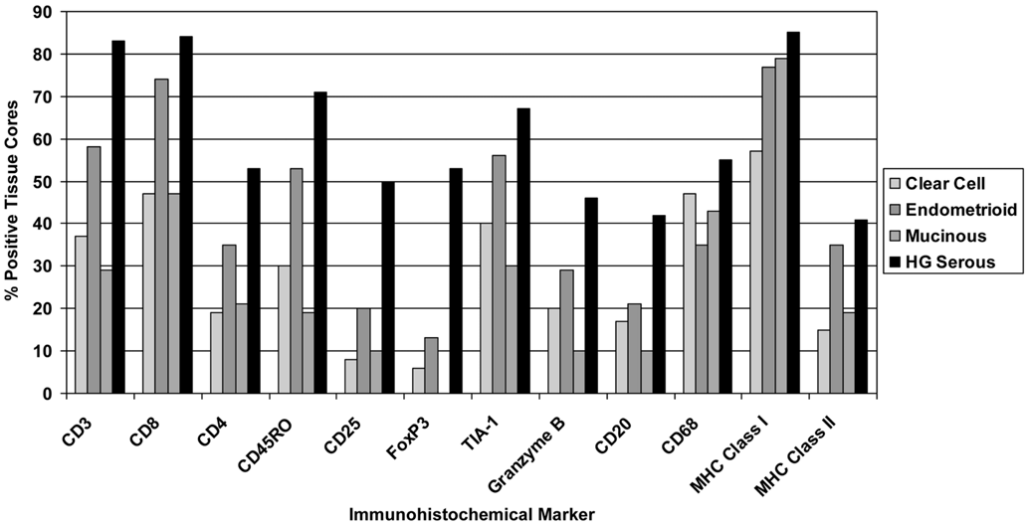
Prevalence of immune infiltrates and other markers across different histologic subtypes of EOC. Bars indicate the percentage of tumors scoring positive for intrapithelial cells expressing CD3, CD8, CD4, CD45RO, CD25, FoxP3, TIA-1, Granzyme B, CD20 and CD68. Expression of MHC class I and II by tumor epithelium is also shown. Data were derived from optimally debulked patients.

In general, immune infiltrates were less prevalent in the other histological subtypes compared to the high-grade serous cases discussed previously. This was true for all lymphocyte markers studied (i.e., CD3, CD8, CD4, CD45RO, CD25, FoxP3, TIA-1, Granzyme B, and CD20) (Fig. 5). The difference was most striking for the markers FoxP3, CD25 and CD20. After the high-grade serous cases, the next highest frequency of immune infiltrates was seen in the endometrioid subtype (Fig. 5).

We examined the association between immune infiltrates and DSS in the endometrioid and clear cell subtypes; the number of mucinous cases was too small to perform robust statistical analysis. For endometrioid cases, the only significant association found was between MHC class II expression and increased DSS (p = 0.039) (data not shown). For clear cell cases, the only significant association found was between the presence of myeloperoxidase-positive infiltrates and decreased DSS (p = 0.040, data not shown). Thus, the relationship between immune infiltrates and survival differs greatly between histological subtypes of EOC.

## Discussion

We systematically examined the relationship between immune infiltrates and patient survival in three large EOC series. In accord with Clarke et al. [12], we found that high-grade serous tumors have a distinct immunological profile compared to the endometrioid, clear cell and mucinous subtypes. Furthermore, we found that immune infiltrates were generally more prevalent in tumors from patients with optimal cytoreduction. FoxP3, TIA-1 and CD20 emerged as novel immunological markers associated with increased patient survival. Our results highlight the importance of histological subtype in the immunobiology of EOC, which may have important implications for the immunotherapy of this family of diseases.

Intraepithelial lymphocytes (i.e., cells expressing CD3, CD4, CD8, FoxP3 or CD20) were more prevalent in high-grade serous cases, followed by endometrioid cases. Moreover, intraepithelial lymphocytes were more prevalent in tumors from optimally debulked patients compared to patients with macroscopic residual disease. A number of biological features of tumors appear to influence the density of lymphocytic infiltrates. (a) T cell infiltrates are positively associated with expression of MHC class I and II by tumor cells (Table 2), as well as MHC class I antigen processing machinery [15]-[17], [67], suggesting that antigen presentation may be an important determinant of T cell infiltration. (b) In accord with this notion, tumors with loss or mutation of the BRCA1 or p53 genes have an increased density of tumor-infiltrating T cells [12], [66]. This suggests that defective DNA repair and the ensuing genomic instability in tumors may lead to the generation of neo-antigens that trigger host T cell responses. (c) Signaling molecules also play a role, as the density of tumor-infiltrating T cells is negatively associated with expression of VEGF, B7-H1/PD-L1 and endothelin B receptor by tumors [5], [11], [68] and positively associated with expression of the chemokines CXCL9, CCL21, CCL22, CCL2 and CCL5 [5], [28], [69]. (d) Finally, two groups have reported gene expression profiles that correlate with the presence of tumor-infiltrating T cells in EOC [12], [70]. These latter studies confirm some of the above associations (e.g., MHC class I and II, beta 2 microglobulin, TAP1 and 2) and identify new factors associated with T cell infiltrates (e.g., IL-15, IL-32 and numerous interferon-induced genes). Presumably one or more of the above factors accounts for the observed enrichment of tumor-infiltrating lymphocytes in high-grade serous and optimally cytoreduced cases.

Although the association between intraepithelial CD8+ T cells and increased survival in EOC is a highly reproducible finding [10]-[17], relatively little is known about the functional phenotype of these CD8+ T cells. Several lines of evidence suggest a classic cytolytic response underlies favorable outcomes. For example, others have reported positive associations between survival and intratumoral expression of IFN- γ [18], [19], the IFN- γ receptor [20], as well as numerous interferon-responsive genes such as MHC class I [24]-[26], MHC class I antigen processing machinery [17], MHC class II [15], [16], and IRF-1 [21]. IL-18 [22] and TNF-α [23] also appear to be important components of the T cell response, as both cytokines are positively associated with survival. We examined two components of cytolytic granules, Granzyme B and TIA-1, both of which showed an association with CD8+ T cell infiltrates. Of these two markers, only TIA-1 showed a statistically significant association with survival in high-grade serous cases (Fig. 4). TIA-1+ cells have also been described in medullary breast cancer [71], [72] and melanoma [73], where they are associated with favorable prognostic features. By contrast, tumor-infiltrating TIA-1+ cells are associated with decreased survival in lymphoma [74]-[78]. Interestingly, TIA-1 is not simply a marker of cytolytic granules; it is an RNA binding protein involved in post-transcriptional mRNA regulation [79]. It remains to be determined whether the association between intraepithelial TIA-1+ cells and survival in EOC is due to the role of this protein in cytolytic granule function or mRNA regulation.

Treg infiltrates have previously been associated with decreased survival in ovarian EOC [10], [27], [28]. However, in the present study and one other recent report [14], FoxP3+ infiltrates were associated with increased survival. These seemingly contradictory findings may be attributable to several factors. First, not all studies take into consideration the histological subtypes of EOC, or the extent of residual disease; in the present study, FoxP3+ cells were only associated with survival in high-grade serous tumors from optimally debulked patients. Second, a variety of antibodies have been used to detect FoxP3, which can lead to discordant results [80]. Third, different scoring criteria may be used. For example, the precise intratumoral location of Tregs is an important determinant of prognosis in gastric cancer [81]. Fourth, the molecular markers used to define Tregs differ between studies. Although FoxP3 is still regarded as the most reliable marker of Tregs in human cancer [82], [83], it can also be expressed by epithelial tumor cells [84]-[86] and *in vitro* activated CD4+ and CD8+ T cells [87]-[95]. For these reasons, some studies include CD25 as a second marker of Tregs [10], [28]. However, like FoxP3, CD25 is potentially expressed by effector T cells, so it is not clear that dual staining for FoxP3 and CD25 more accurately identifies Tregs [89], [96]. Other characteristics of Tregs include high expression of GITR and CTLA-4 and low expression of CD127 and CD49d and [97], [98], however these markers are technically difficult to assess on paraffin-embedded TMAs.

These technical considerations notwithstanding, there is mounting evidence that tumor-infiltrating FoxP3+ cells are associated with a favorable prognosis in EOC, colorectal cancer, head and neck cancer, and lymphoma [14,33,-36,99-102]. How might FoxP3+ T cells promote favorable outcomes? In the present study, FoxP3+ cells were strongly associated with other effector T cells, and similar results have been reported in melanoma [103]. Thus, FoxP3+ cells may simply be an indicator of a strong CD8+ T cell response, which might outweigh any immunosuppressive effects of FoxP3+ cells. Alternatively, subsets of human FoxP3+ T cells have recently been shown to have a pro-inflammatory, IL-17-producing phenotype [104]-[106]. Indeed, CD4+ T cells can be skewed toward this so-called Th17 phenotype by exposure to TGF-β in combination with IL-6, IL-1 or IL-23 [107]-[109]. These factors are present in the EOC tumor environment [8], and accordingly, Th17 cells have been reported in EOC [110]-[112]. Thus, the association between FoxP3+ cells and increased survival could potentially reflect an underlying Th17-like anti-tumor response. Clearly, more work is required to determine the extent to which FoxP3+ T cells in EOC represent Tregs versus Th17 or other effector T cells.

The observation that intraepithelial CD20+ infiltrates are associated with increased DSS is a novel finding in EOC. Dong et. al. reported that B cells in ascites were associated with shorter survival in EOC [113], however their study focused on B cells in peritoneal and pleural effusions collected after chemotherapy, which by definition constitutes a poor outcome cohort. Indeed, in the present study, intraepithelial CD20+ B cells showed no association with survival in patients with high-risk, suboptimally debulked disease. Tumor-infiltrating CD20+ B cells are a hallmark of medullary breast cancer and have been proposed to mediate a favorable prognosis [114], [115]. Moreover, the presence of a B cell transcriptional signature in node-negative breast cancer is associated with increased survival [116]. B cell infiltrates in breast cancer represent clonally expanded populations, express somatically hypermutated IgG molecules, and recognize target antigens such as ganglioside D3 and surface-translocated actin [114], [115], [117]-[120]. It is unclear how tumor-infiltrating B cells promote favorable outcomes in cancer. In theory, their actions could be mediated by secreted antibodies, which can promote the opsonization of tumor antigens, complement-mediated destruction of tumor cells, or antibody-dependent cellular cytotoxicity. Apart from producing antibodies, B cells can also present antigen to both CD4+ and CD8+ T cells [121]-[131]. In this regard, it is noteworthy that ovarian tumors show low numbers of CD1a+ dendritic cells; perhaps CD20+ B cells serve as alternative antigen presenting cells in the tumor environment. This latter idea fits well with the observed co-localization of tumor-infiltrating B cells and CD8+ T cells in EOC, as well as in medullary breast cancer, non-small cell lung cancer and cervical cancer [72], [132]-[134].

The presence of macrophages has been associated with poor prognosis in various human cancers [135], [136]. However, in accord with a prior report by Shah et. al. [66], we found no association between CD68+ infiltrates and survival in EOC. Importantly, however, CD68 is not a perfect marker of macrophages, as it is also expressed by dendritic cells and some non-myeloid cells [137]. Furthermore, CD68 does not distinguish between macrophages polarized towards the pro-inflammatory (M1) or tumor-promoting (M2) phenotypes. M1 macrophages have the capacity to kill tumor cells, whereas M2 macrophages promote tissue repair and angiogenesis [136]. Similarly, an immunosuppressive subpopulation of macrophages has been described in EOC based on expression of the signaling molecule B7-H4 [52]. Thus, additional functional markers may be required to fully define the role of macrophages in the immunobiology of EOC.

While this study focused on the relationship between immune infiltrates and prognosis after standard treatments, the results may also inform the design of immunotherapies for EOC. First, our findings suggest that high-grade serous tumors may be especially sensitive to T cell responses. Second, our data indicates that, in patients with residual disease, the influence of T cells may be overwhelmed by other factors. Third, the positive association between intraepithelial FoxP3+ cells and survival reported here and previously [14] prompts a reconsideration of strategies to deplete regulatory T cells from EOC patients. And fourth, the association between intraepithelial CD20+ cells and survival suggests the humoral immune response may play an important role in anti-tumor immunity that could be exploited therapeutically in parallel with CD8+ T cell responses.

## Materials and Methods

### Study subjects

All specimens and clinical data were obtained with informed written consent under protocols approved by the Research Ethics Board of the BC Cancer Agency and the University of British Columbia. The main cohort used for this study consisted of 199 women with high-grade serous ovarian cancer seen at the BC Cancer Agency from 1984 to 2000 (OvCaRe Ovarian Tumour Bank, Vancouver, BC, Canada). Tumor tissue was obtained at the time of primary surgery prior to any other treatment. Patients had no macroscopic residual disease following surgical debulking. All patients underwent standard treatment consisting of surgery followed by standard platinum-based chemotherapy. Table 1 shows the general clinical characteristics of the 199-case cohort. We also analyzed a second cohort of mucinous (n = 31), endometrioid (n = 125) and clear cell (n = 132) EOC cases. Patients in this cohort were also diagnosed from 1984 to 2000, were optimally cytoreduced, and received platinum-based chemotherapy. Finally, we analyzed a third cohort of 220 high-grade serous EOC patients categorized as extreme risk due to the presence of residual macroscopic disease. Patients in this cohort were treated from 1996 to 2000 and received platinum-based chemotherapy.

### Tumor specimens

Tumor tissue was obtained during primary cytoreductive surgery, fixed in 10% neutral buffered formalin, processed using standard procedures and embedded in paraffin. A tissue microarray (TMA) was constructed by taking duplicate 0.6 mm cores from representative regions of each tumor block after review of hematoxylin- and eosin-stained sections by a pathologist. TMAs were assembled using a Pathology Devices tissue arrayer (Westminster, MD).

### Immunohistochemistry

Immunohistochemistry for CD20, CD3, CD4, CD8 and Granzyme B was performed as described in Clarke et al.[12]. The remaining unstained slides were received at the Trev and Joyce Deeley Research Centre where immunohistochemistry was performed for CD45RO, TIA-1, FoxP3, CD25, OX-40, CD56, CD57, CD1a, CD208, myeloperoxidase, CD68, COX-2, MHC Class I and MHC Class II. Following deparaffinization, the slides were placed in a Ventana Discovery XT autostainer (Ventana, Tucson, AZ) for immunohistochemical staining. Ventana's standard CC1 protocol was used for antigen retrieval. Primary antibodies are listed in Table 3.

**Table 3:** Primary antibodies used for immunohistochemistry.

Antigen	Clone	Supplier	Source	Concentration
CD3	Polyclonal	Cell Marque	Rabbit	1/300
CD8	C8/144C	DAKO	Rabbit	1/50
CD4	4B12	Novocastra	Mouse	1/50
Granzyme B	GrB-7	DAKO	Mouse	1/25
CD45RO	UCHL-1	Lab Vision	Mouse	1/300
TIA-1	TIA-1	Abcam	Mouse	1/50
FoxP3	eBio7979	eBioscience	Mouse	1/50
CD25	4C9	Lab Vision	Mouse	1/40
OX-40 (CD134)	ACT35	BD Pharmingen	Mouse	1/50
CD20	L26	DAKO	Mouse	1/250
CD56	123C3.D5	Lab Vision	Mouse	1/50
CD57	NK1	Lab Vision	Mouse	1/200
CD1a	O10	Lab Vision	Mouse	1/50
CD208	1010E1.01	Imgenix	Rat	1/50
Myeloperoxidase	Polyclonal, Catalogue # RB-373	Lab Vision	Rabbit	1/200
CD68	PG-M1	Lab Vision	Mouse	1/50
Cyclooxygenase-2 (COX-2)	SP21	Cell Marque	Rabbit	1/10
MHC class I (A, B, C)	EMR8-5	MBL	Mouse	1/500
MHC class II (DR, DP & DQ)	CR3/43	Affinty BioReagents	Mouse	1/50

TMAs were incubated with primary antibodies for 60 minutes at room temperature, and the appropriate cross-adsorbed, biotinylated secondary antibody (Jackson Immunoresearch, West Grove, PA) was applied for 32 minutes. Bound antibodies were detected using the DABMap kit (Ventana), counterstained with hematoxylin (Ventana), and coverslipped manually with Cytoseal-60 (Richard Allan, Kalamazoo, MI).

### Histopathological analysis

Immunostained TMAs were examined by a pathologist and scored using a variety of methods depending on the marker studied. For CD20, CD8, CD4, CD45RO, TIA-1, Granzyme B, CD25, OX40, CD1a, CD56, CD57, and myeloperoxidase, only cells residing within the epithelial compartment of the tumor were counted. FoxP3 was similarly scored within the epithelium but a stromal score was also obtained. Tumors were scored as 0 (no cells), 1 (1-5 cells), 2 (6-19 cells) or 3 (20+ cells); results were binarized as positive (IHC score 1, 2, or 3) or negative (IHC score 0). CD3 was scored as 0 (no cells present), 1 (cells present in stroma only), 2 (cells present in the epithelial compartment) or 3 (cells present in both the epithelial and stromal regions of the tumor); scores of 0 and 1 were reported as negative while a score of 2 or 3 was reported as positive. CD68 was scored as 0 (no cells present), 1 (luminal or stromal cells), 2 (scattered, <20 intraepithelial cells), or 3 (>20 intraepithelial cells), results were binarized in the same manner as CD3. COX-2 was scored as 0 (negative), 1 (equivocal, 0-1%), 2 (patchy, >1% to 50%) or 3 (diffuse, >50%) scores of 0 and 1 were reported as negative and scores of 2 or 3 were reported as positive. For MHC class I and II, samples were scored as 0 (negative), 1 (focal, <10%), 2 (patchy, 10-50%) or 3 (diffuse, >50%). Scores of 0, 1 or 2 were reported as negative and scores of 3 were reported as positive.

### Statistical analysis

Statistical analysis was performed using JMP statistical software (v7.0) (SAS Institute, Cary, NC). Univariate analysis was carried out using the Chi-Squared statistic. The log-rank test was used to compare Kaplan-Meier curves. *p*-values less than 0.05 were considered significant.
