# Correction: Systematic Analysis of Immune Infiltrates in High-Grade Serous Ovarian Cancer Reveals CD20, FoxP3 and TIA-1 as Positive Prognostic Factors

**DOI:** 10.1371/annotation/976c923b-d991-4a33-b060-cdd8770bdf5d

**Published:** 2013-07-23

**Authors:** Katy Milne, Martin Köbel, Steven E. Kalloger, Rebecca O. Barnes, Dongxia Gao, C. Blake Gilks, Peter H. Watson, Brad H. Nelson

All specimens and clinical data were obtained with either informed written consent or a formal waiver of consent under protocols approved by the Research Ethics Board of the BC Cancer Agency and the University of British Columbia. 

